# The relationship between obstetrical interventions and the increase in U.S. preterm births, 2014-2019

**DOI:** 10.1371/journal.pone.0265146

**Published:** 2022-03-30

**Authors:** Marian F. MacDorman, Marie Thoma, Eugene Declercq, Elizabeth A. Howell

**Affiliations:** 1 Maryland Population Research Center, University of Maryland, College Park, Maryland, United States of America; 2 Department of Family Science, University of Maryland School of Public Health, College Park, Maryland, United States of America; 3 Department of Community Health Sciences, Boston University School of Public Health, Boston, Massachusetts, United States of America; 4 Department of Obstetrics and Gynecology, Perlman School of Medicine, University of Pennsylvania, Philadelphia, Pennsylvania, United States of America; Texas A&M University College Station, UNITED STATES

## Abstract

We examined the relationship between obstetrical intervention and preterm birth in the United States between 2014 and 2019. This observational study analyzed 2014–2019 US birth data to assess changes in preterm birth, cesarean delivery, induction of labor, and associated risks. Logistic regression modeled the odds of preterm obstetrical intervention (no labor cesarean or induction) after risk adjustment. The percentage of singleton preterm births in the United States increased by 9.4% from 2014–2019. The percent of singleton, preterm births delivered by cesarean increased by 6.0%, while the percent with induction of labor increased by 39.1%. The percentage of singleton preterm births where obstetrical intervention (no labor cesarean or induction) potentially impacted the gestational age at delivery increased from 47.6% in 2014 to 54.9% in 2019. Preterm interventions were 13% more likely overall in 2019 compared to 2014 and 17% more likely among late preterm births, after controlling for demographic and medical risk factors. Compared to non-Hispanic White women, Non-Hispanic Black women had a higher risk of preterm obstetric interventions. Preterm infants have higher morbidity and mortality rates than term infants, thus any increase in the preterm birth rate is concerning. A renewed effort to understand the trends in preterm interventions is needed to ensure that obstetrical interventions are evidence-based and are limited to those cases where they optimize outcomes for both mothers and babies.

## Introduction

Preterm birth (birth before 37 completed weeks of gestation) has long been a critical measure of infant health. After a decline from 2006–2014, the United States preterm birth rate increased from 9.57% of U.S. births in 2014 to 10.23% in 2019 [1]. The increase was larger among singleton (9.4%) than among multiple (3.1%) births [2]. An increase in the preterm birth rate is of concern because rates of death and disability are higher among preterm infants than among infants born at term (37–41 weeks) [3, 4]. In 2018, the infant mortality rate for late preterm (34–36 weeks of gestation) infants (8.21 infant deaths per 1,000 live births) was 4 times the rate for term infants (2.05), while the mortality rate for infants born before 34 weeks of gestation (115.39), was 56 times the rate for term infants [5].

One study estimated the annual cost of preterm birth as $25 billion in 2016 [6]. Factors that have been found related to the preterm birth rate include maternal stress [7], air pollution [8], rural residential area [9], young maternal age [10] and social determinants [11]. Proposed remedies for the problem have ranged from social programs [12] to low dose aspirin [13], precision medicine [14] and progesterone vaginal tablets [15].

An earlier paper explored the role obstetrical interventions might have played in the 1991–2006 rising trend in preterm births and found much of the increase associated with increasing use of preterm cesarean section and induction or labor [16]. The purpose of this paper is to revisit the relationship between changes in the use of obstetrical interventions and the recent rise in the preterm birth rate in the United States between 2014 and 2019. Specifically, we explored trends in singleton preterm births, delivery methods (cesarean or vaginal, with or without labor), and induction of labor.

## Methods

We analyzed data from the National Center for Health Statistics natality data files from 2014–2019. These files contain detailed information on each of the nearly 4 million births in the United States each year [17]. Gestational age was measured using the obstetric estimate of gestation, defined as “the best obstetric estimate of the infant’s gestation in completed weeks based on the birth attendant’s final estimate of gestation.” [18].

We examined trends in singleton preterm births and in cesarean and induction of labor among singleton, preterm births overall and by demographic and medical variables (Tables 1 and 2). Variables were categorized using the categories shown in Table 1. Race and ethnicity was self-reported by the birthing woman. Since not all states had data on all items, for 2014, states were dropped for the specific variable only as follows: Single race of mother: New Jersey and Rhode Island; body mass index, and method of payment for delivery: New Jersey, Rhode Island and Connecticut. In addition, although maternal education, diabetes, and hypertension were reported by all states, non-comparable data for New Jersey, Rhode Island and Connecticut were dropped for 2014. A two-proportion z test was used to assess statistical significance and all text statements that a given measure was higher or lower than another indicates statistical significance at the p < .05 level.

**Table 1:** Percent of singleton births that were preterm by selected characteristics, United States, 2014 and 2019.

Characteristics			Percent change
	2014	2019	2014–2019
Race/ethnicity			
Total	7.73	8.46	9.4
Hispanic	7.68	8.56	11.5
Non-Hispanic			
White	6.91	7.44	7.7
Black	11.24	12.12	7.8
Native American	9.12	10.27	12.6
Asian	6.65	7.32	10.1
Pacific Islander	9.61	10.02	4.3
Multiracial	8.12	9.00	10.8
Gestational age (weeks)			
<37	7.73	8.46	9.4
<34	2.07	2.14	3.4
34–36	5.66	6.32	11.7
Maternal age			
<20	8.84	9.36	5.9
20–24	7.84	8.46	7.9
25–29	7.2	7.89	9.6
30–34	7.23	7.92	9.5
35–39	8.53	9.47	11.0
40–44	10.59	11.84	11.8
45+	14.19	15.33	8.0
Maternal education			
High school or less	8.83	9.70	9.9
Some college	7.98	8.91	11.7
Bachelor’s degree or greater	5.96	6.56	10.1
Live birth order			
1	7.84	8.52	8.7
2	6.7	7.27	8.5
3	7.78	8.52	9.5
4+	9.95	11.16	12.2
Body Mass Index			
Underweight (<25)	9.47	10.05	6.1
Normal weight (25–29.9)	6.99	7.4	5.9
Overweight (30–34.9)	7.37	7.91	7.3
Obese (35+)	8.83	9.93	12.5
Payment for delivery			
Medicaid	8.82	9.83	11.5
Private insurance	6.76	7.40	9.5
Other	7.57	7.78	2.8
Diabetes			
Pre-pregnancy	23.57	26.96	14.4
Gestational	10.94	11.75	7.4
Hypertensive disease			
Pre-pregnancy	20.20	21.93	8.6
Gestational or eclampsia	20.51	19.70	-3.9

Note: Not stated responses were dropped before percentages were computed. Race data are for women reporting a single race. 2014 data excludes New Jersey and Rhode Island which did not report single/multiple race data in 2014. Diabetes, hypertension and method of payment for delivery were not reported in New Jersey, Rhode Island, and Connecticut in 2014.

**Table 2:** Percentage of singleton preterm births with cesarean or induction of labor by gestational age and maternal race/ethnicity, United States, 2014–2019.

	Preterm		
	2014	2019	% change
Cesarean			
Total	43.2	45.8	6.0
Gestational age			
Early preterm	54.4	58.0	6.6
Late preterm	39.1	41.7	6.6
Race/ethnicity			
Hispanic	43.0	44.5	3.5
Non-Hispanic			
White	43.2	46.0	6.6
Black	44.4	48.4	9.0
Native American	40.8	42.0	3.0
Asian	41.0	43.2	5.3
Pacific Islander	40.7	41.6	2.2
Multiple Race	39.6	42.5	7.3
Induction			
Total	13.6	18.9	39.1
Gestational age			
Early preterm	8.1	10.0	22.9
Late preterm	15.6	21.9	40.4
Race/ethnicity			
Hispanic	12.1	17.7	46.5
Non-Hispanic			
White	14.4	20.2	40.3
Black	13.8	18.0	30.7
Native American	14.2	21.6	51.9
Asian	11.7	16.5	41.0
Pacific Islander	11.9	13.6	14.3
Multiple Race	15.3	20.0	30.7

Note: Race data shown are for women reporting a single race. 2014 data excludes New Jersey and Rhode Island which did not report single/multiple race data in 2014.

The method of delivery (vaginal or cesarean), induction of labor (yes, no), and “If cesarean, was a trial of labor attempted?” (yes, no) variables available from the birth certificate [19] were used to group births into five separate categories: spontaneous vaginal, cesarean with labor, no labor cesarean, induced vaginal, and cesarean after attempted induction. The purpose of this analysis was to estimate the proportion of singleton preterm births in which obstetrical intervention may have affected the gestational age at delivery. If a woman already in labor has a preterm cesarean delivery, it is unlikely to substantially affect the infant’s gestational age, given that she would probably have delivered in a short time regardless of the intervention. However, if a woman not in labor has a preterm cesarean delivery or induction of labor, it may affect gestational age at delivery because it is unknown how much longer the pregnancy might have continued without the intervention [16]. Cesarean births with no reported labor were defined as births in which the trial of labor variable was marked “no”. We combined this cesarean-without-labor category with inductions to yield an estimate of the percentage of singleton preterm (and late preterm) births in the US where obstetrical intervention may have affected the gestational age at delivery.

Logistic regression was used to examine the odds of preterm obstetrical intervention (induction or no labor cesarean) among all singleton births in 2014 and 2019, with 2014 as the reference year. Three models were run with variable categories as shown in Table 3. Model 1 adjusted for race and ethnicity. Model 2 adjusted for race/ethnicity, maternal age, education, live birth order, body mass index, and method of payment for the delivery. Model 3 adjusted for all variables in model 2 plus preexisting or gestational diabetes and preexisting or gestational hypertensive disease. Covariates in the model were selected based on their potential relationship with preterm birth and/or obstetric intervention. We computed adjusted odds ratios separately for the total preterm and late preterm categories. For the preterm category, preterm births were compared to all births regardless of gestational age. For the late preterm category, late preterm births were compared to all late preterm and term births. Records with missing values (<2.5% for all variables) were excluded from the models. As the study was based on de-identified government public-use data sets that do not contain any identifiable private information, IRB approval was not required.

**Table 3:** Odds ratios for preterm obstetrical intervention (no labor cesarean or induction of labor) by race/ethnicity, United States, 2014 and 2019.

**Preterm**			
	Model 1, OR (95% CI)	Model 2, OR (95% CI)	Model 3, OR (95% CI)
Year			
2014	Reference	Reference	Reference
2019	1.28 (1.27, 1.29)	1.22 (1.21, 1.23)	1.12 (1.12, 1.13)
Race/ethnicity			
Hispanic	1.09 (1.08, 1.10)	0.99 (0.98, 1.00)	1.07 (1.06, 1.08)
Non-Hispanic			
White	Reference	Reference	Reference
Black	1.65 (1.63, 1.66)	1.47 (1.46, 1.49)	1.42 (1.41, 1.44)
Native American	1.31 (1.26, 1.36)	1.17 (1.13, 1.22)	1.07 (1.02, 1.11)
Asian	0.89 (0.87, 0.90)	0.92 (0.91, 0.94)	0.98 (0.96, 1.00)
Pacific Islander	1.23 (1.15, 1.32)	1.00 (0.93, 1.08)	1.04 (0.96, 1.12)
Multiracial	1.13 (1.11, 1.16)	1.09 (1.06, 1.11)	1.10 (1.07, 1.12)
**Late preterm**			
	Model 1, OR (95% CI)	Model 2, OR (95% CI)	Model 3, OR (95% CI)
Year			
2014	Reference	Reference	Reference
2019	1.33 (1.32–1.34)	1.27 (1.26, 1.28)	1.17 (1.16, 1.18)
Race/ethnicity			
Hispanic	1.05 (1.04, 1.07)	0.97 (0.95, 0.98)	1.04 (1.02, 1.05)
Non-Hispanic			
White	Reference	Reference	Reference
Black	1.42 (1.41–1.44)	1.28 (1.26, 1.30)	1.24 (1.22, 1.26)
Native American	1.30 (1.24–1.36)	1.17 (1.12, 1.22)	1.06 (1.01, 1.10)
Asian	0.88 (0.86–0.89)	0.92 (0.90, 0.94)	0.96 (0.94, 0.98)
Pacific Islander	1.15 (1.06–1.25)	0.93 (0.85, 1.01)	0.95 (0.87, 1.05)
Multiracial	1.06 (1.04–1.09)	1.04 (1.01, 1.07)	1.05 (1.02, 1.08)

Model 1 adjusted for race/ethnicity. Model 2 adjusted for race/ethnicity, maternal age, education, live birth order, obesity, and method of payment for delivery. Model 3 adjusted for variables in model 2 plus diabetes and hypertensive disorders.

## Results

In 2014, there were 297,460 singleton preterm births out of 3,848,214 singleton births at any gestational age registered in the United States. In 2019, the numbers were 306,646 and 3,623,963, respectively. Numbers were similar from 2015–2018.

The overall proportion of preterm births in the United States increased from 9.57% in 2014 to 10.23% by 2019, a percentage increase of 6.9%. Among our study population of singleton births, the increase between 2014 (7.73%) and 2019 (8.46%) was 9.4% (Table 1). The percent of singleton, late preterm births increased from 5.66% of all singleton births in 2014 to 6.32% in 2019 –an increase of 11.7%. The increase was much smaller (3.4%) for births that occurred earlier in pregnancy. The increase in late preterm births accounted for 90.4% of the total increase in preterm births from 2014–2019.

When examined by race/ethnicity, we found that increases in singleton preterm births were largest for Native American (12.6%) and Hispanic (11.5%) women, and moderate for non-Hispanic Black (7.8%) and non-Hispanic White women (7.7%). Compared to non-Hispanic White women, the 2019 singleton preterm birth rate was 62.9% higher for non-Hispanic Black women, 38.0% higher for Native American women, and 34.6% higher for Pacific Islander women. Increases were also larger for women aged 35–44, those with 4 or more prior births, were obese, had pre-pregnancy diabetes, or whose births were paid for by Medicaid.

**Fig 1:**
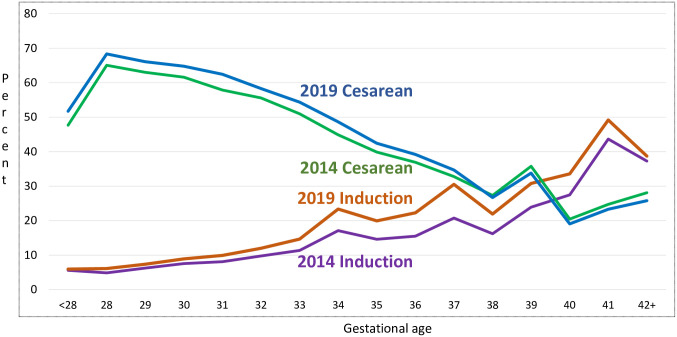
Percentage of singleton births with cesarean or induction by gestational age, United States, 2014 and 2019.

The percentage of singleton preterm births delivered by cesarean section increased from 43.2% in 2014 to 45.8% in 2019 –an increase of 6.0% (Table 2). Increases were similar for early and late preterm births. In 2019, non-Hispanic Black women had the highest percentage of singleton preterm births delivered by cesarean (48.4%), followed by non-Hispanic White (46.0%) and Hispanic (44.5%) women. Increases in preterm births delivered by Cesarean from 2014–2019 were largest for non-Hispanic Black women (9.0%). When examined by detailed gestational age, we found that the percentage of cesarean births were generally higher in 2019 than in 2014 in the preterm categories, and lower in the term birth gestational age categories (Fig 1).

The percentage of all singleton preterm births with induction of labor increased substantially from 13.6% in 2014 to 18.9% in 2019 –an increase of 39.1% (Table 2). Compared to early preterm births, increases were larger among late preterm births where induction rates among singleton births averaged 21.9% in 2019 compared to 15.6% in 2014, an increase of 40.4% in a five-year period. When examined by race and ethnicity, we found that the largest increases were for Native American (51.9%), Hispanic (46.5%), Asian (41.0%) and non-Hispanic White (40.3%) women. When examined by detailed gestational age, inductions were higher in 2019 than in 2014 at every gestational age, with the largest differences among late preterm and early term births (Fig 1).

**Fig 2:**
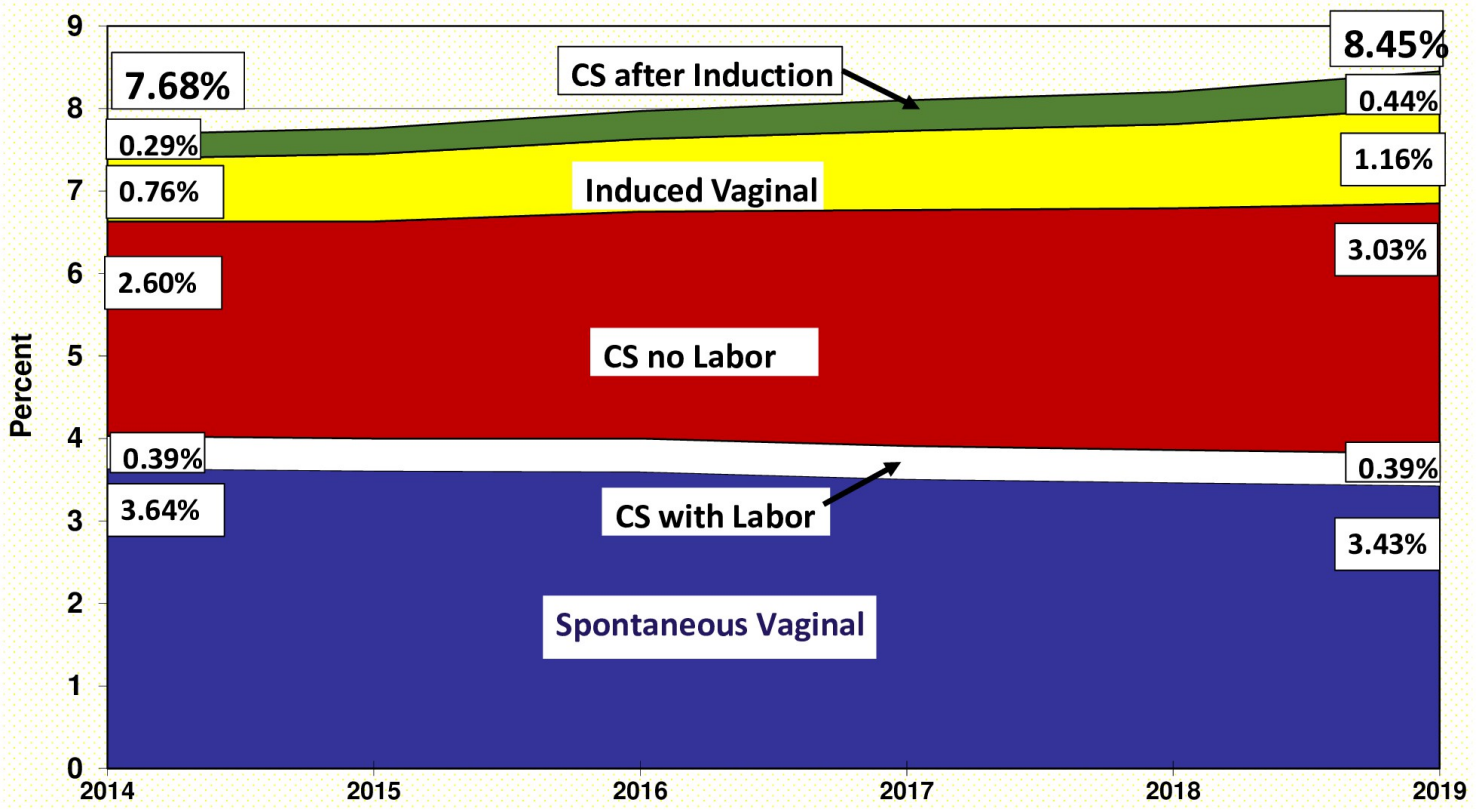
Percentage of singleton preterm births, out of total births, by method of delivery, United States, 2014–2019.

In Fig 2, the overall percentage of singleton preterm births is divided into subgroups based on method of delivery: spontaneous vaginal, cesarean with labor, no labor cesarean, induced vaginal, and cesarean after attempted induction. The latter 3 categories represent births where obstetrical intervention had the potential to impact the gestational age at birth. The percentage of singleton births that were preterm among spontaneous vaginal deliveries declined by 5.8% from 3.64% in 2014 to 3.43% in 2019. In contrast, the percentage of singleton preterm births with a no labor cesarean rose from 2.60% in 2014 to 3.03% in 2019, a 16.5% relative increase. For induction of labor, the increase was much larger. The percentage of singleton preterm births with induction of labor (whether ultimately delivered vaginally or by cesarean) increased by 52.3%, from 2014 (1.05%) to 2019 (1.60%).

Next, we examined births by method of delivery as a proportion of all singleton preterm births. We found that the two categories that did not involve interventions that might alter gestational age (spontaneous vaginal births and cesareans with labor) declined from 52.5% to 45.2% of all singleton preterm births (Fig 2). Together, the percentage of singleton, preterm births where obstetrical intervention potentially impacted the gestational age at delivery increased from 47.5% in 2014 to 54.8% in 2019.

**Fig 3:**
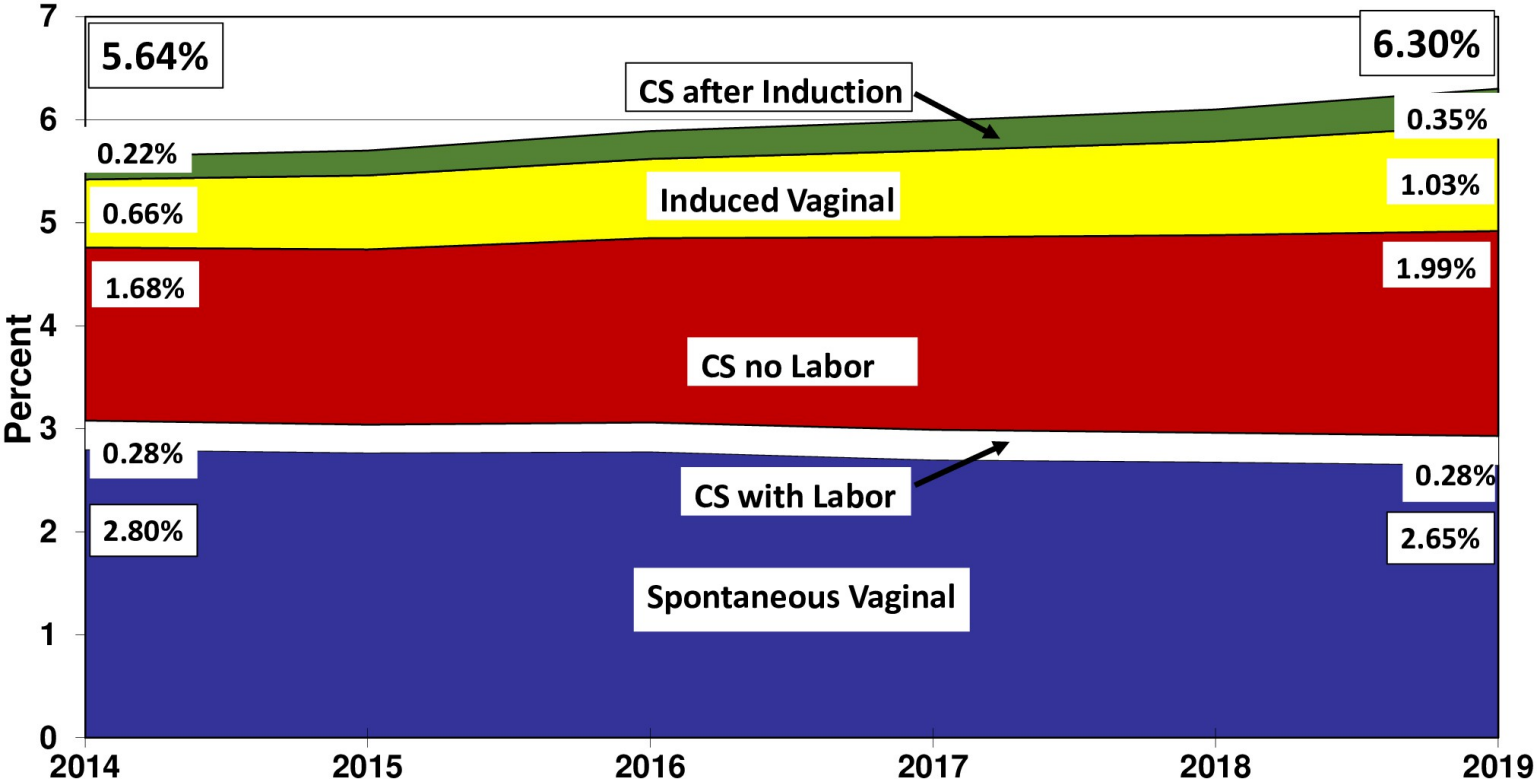
Percentage of singleton, late preterm births, out of total births, by method of delivery, United States, 2014–2019.

When late preterm births were examined as a proportion of total births, we found that the percent of singleton late preterm births with spontaneous vaginal delivery declined by 5.4% from 2014 (2.80%) to 2019 (2.65%) (Fig 3). In contrast, the percentage of singleton late preterm births with no labor cesarean rose from 1.68% in 2014 to 1.99% in 2019, an increase of 19.9%. For induction of labor, the increase was once again much larger. The percentage of singleton late preterm births with induction of labor increased by 57%, from 0.88% in 2014 to 1.38% in 2019.

When examined as a proportion of all late preterm births, we found that the two categories that did not involve interventions that might alter gestational age (spontaneous vaginal births and cesareans with labor) declined from 54.6% of total late preterm births in 2014 to 46.5% in 2019. Conversely, the percentage of singleton late preterm births where obstetrical intervention potentially impacted the gestational age at delivery increased from 45.4% in 2014 to 53.5% in 2019.

Adjusted odds ratios for the likelihood of preterm obstetrical intervention (no labor cesarean or induction of labor) from 2014–2019, with 2014 as the reference category, are presented in Table 3. In the case of all preterm births, when race and ethnicity was controlled for (Model 1), the odds of having a preterm obstetrical intervention were 28% higher in 2019 than in 2014. When socio-demographic characteristics were also controlled for (Model 2), the odds were 22% higher in 2019 than in 2014. And when demographic and medical characteristics were controlled for (Model 3), the odds of having an obstetrical intervention were 13% higher in 2019 than in 2014. In the case of late preterm births, results were similar, but the odds ratios were larger: 33% higher in model 1, 27% in model 2 and 17% in model 3. It should be noted that the odds of having a preterm obstetrical intervention were substantially higher for non-Hispanic Black women than for women of other races/ethnicities in all models, even after controlling for an array of demographic and medical risks.

## Discussion

The percentage of singleton preterm births increased by 9.4% from 2014–2019, and much of this increase involved increases in obstetrical intervention. In a period when the overall preterm rate was increasing, the preterm rate for spontaneous vaginal births *decreased* by 5.8% and there was no change in cesareans following a trial of labor, meaning all of the increase in preterm births was associated with labor interventions. More than half (56%) of that increase was associated with inductions of labor, either in vaginal or cesarean births, while the remainder involved cesareans with no labor. Both preterm cesareans and preterm inductions have increased between 2014 and 2019, though in the case of inductions, the increases were much larger. While the percent of singleton preterm births with cesarean section increased by 16.1%, the percent with induction of labor increased by 52.3%, with more than one in five (21.9%) deliveries among late preterm (34–36 weeks) births now involving an induction. While overall cesarean rates have remained steady since 2007, induction rates, which also remained steady from 2008 to 2014 at 27%, began a steady climb in 2014 and have now reached 29.4% of all births and 36.6% among planned vaginal births (vaginal births and cesareans with a trial of labor) in 2019 [2].

More than one half (54.9%) of singleton preterm births in 2019 had an obstetrical intervention that could have affected the gestational age at delivery. Preterm inductions and cesareans save lives if the circumstances necessitate the procedure. The 2021 ACOG Committee Opinion on Medically Indicated Late-Preterm and Early-Term Deliveries provides an excellent summary of the indications which may necessitate preterm cesarean or induction [20]. However, overuse of these procedures can cause excess morbidity and mortality since preterm infants have substantially worse outcomes than those born at term [5, 6]. Vital statistics data are not sufficient to disentangle all indications for preterm induction or cesarean. However, they are useful for tracking of national trends, and when preterm rates increase nationally, as they have from 2014–2019, it is important to analyze the components of preterm birth, and to begin to look at the reasons for the increase.

From 2009–2014, The March of Dimes and other organizations ran the “Think 39” campaign to prevent non-medically indicated cesareans and inductions prior to 39 weeks gestation [21, 22]. The campaign was spurred in part by prior research showing the role interventions had played in the earlier rise in prematurity rates [16, 23] and it perhaps played a role in reversing the earlier rise in prematurity, which peaked in 2006 and began a steady decline until 2014. More recently, the Joint Commission National Quality Measures added a measure for the number of births with elective cesareans or inductions from 37–39 weeks’ gestation [24]. This measure, while useful, does not directly address preterm inductions or cesareans.

Preterm interventions were 13% more likely overall in 2019 compared to 2014 and 17% more likely among late preterm births, after controlling for demographic and medical risk factors. Some of the rise in preterm interventions during this period may be associated with increasing rates of chronic illness, including hypertension among women, although we did control for hypertensive disorders in the multivariate modeling. For example, data indicate increasing rates of preeclampsia, which is a leading indication for preterm intervention [25, 26]. Additionally, concern with rising rates of maternal mortality and morbidity, may lead to more aggressive management of delivery in an effort to optimize both maternal and infant outcomes.

While increases in rates of preterm births were relatively similar among White and Black women from 2014 to 2019, we did find overall disparities in use of preterm interventions by race and ethnicity. After controlling for a wide range of demographic and medical risk factors, including obesity, diabetes and hypertension, non-Hispanic Black women were 42% more likely to receive an obstetrical intervention prior to 37 weeks gestation and 24% more likely between 34 and 36 weeks gestation than were White women. Our finding of higher use of obstetrical intervention in preterm deliveries to non-Hispanic Black women is consistent with prior research documenting higher rates of cesarean deliveries for Black women overall [1]. Black women have higher rates of pregnancy complications, maternal morbidity and mortality [27–30] and higher rates of preterm interventions may reflect more aggressive management to optimize maternal outcomes. In addition, growing evidence suggests that both implicit and explicit bias impact delivery [30, 31]. Whether differential treatment drives some of the elevated rates of preterm birth interventions among Black women is unclear. In the setting of term pregnancies, investigators have found that standardization of hospital protocols for labor induction reduces disparities and improves maternal and neonatal outcomes among Black women. [32].

### Strengths and limitations

The strengths of the study include the comprehensive population-based data set which included all births in the United States for a given year together with many demographic and medical variables. Limitations include concerns about the accuracy of reporting of some items on the birth certificate. Most demographic items and some medical items (maternal age, race/ethnicity, live birth order, method of delivery, payment for delivery, gestational age), are considered to be well reported on birth certificates [33–36]. Other variables such as induction of labor, diabetes, and hypertensive disorders tend to be underreported on birth certificates [33¸^34^, ^37^–^39^]. In addition, birth certificate data do not capture indication for preterm birth, which would allow for a more comprehensive assessment of trends in preterm intervention. Also, while we controlled for a wide range of demographic and medical conditions, the racial and ethnic differences we found may be the result of other factors not captured in our dataset.

## Conclusions

The United States prematurity rate increased by 20% between 1990 and 2006 [40]. After the considerable positive effect of campaigns to reduce prematurity that saw the prematurity rate decline 11.8% between 2006 and 2014, the U.S. has experienced another trend toward increasing rates of prematurity. Notably, however, as in the earlier period of rising rates, prematurity among spontaneous vaginal births has declined while the overall rate has increased. All of the increase since 2014 is associated with pre-labor cesareans and inductions. One of the primary remedies for the earlier crisis was a national effort to reduce primary cesareans and non-medically indicated inductions. Our findings suggest that the latest increase is more strongly related to inductions and raises the question of why inductions prior to 37 weeks are rising so rapidly. Such a shift could be the result of a deteriorating medical risk profile for U.S. women, with inductions an appropriate step to mitigate rising trends in maternal morbidity and optimize outcomes for the maternal infant dyad, or it could reflect a cultural predisposition to intervene in some maternity units [41]. In addition to exploring biomedical reasons for prematurity, there needs to be a renewed effort to understand the trends in preterm interventions to ensure that obstetrical interventions are evidence-based, and are limited to those cases where they optimize outcomes for both mothers and babies.

## References

[1] Martin JA, Hamilton BE, Osterman MJK, Driscoll AK. Births: Final data for 2019. National vital statistics reports vol 70 no 2. Hyattsville, MD: National Center for Health Statistics. 2021. 33814033

[2] Centers for Disease Control and Prevention. CDC Wonder online databases. https://wonder.cdc.gov/ Accessed: August 1, 2021.

[3] Di Renzo GC, Tosto V, Giardina I. The biological basis and prevention of preterm birth. *Best Pract Res Clin Obstet Gynaecol*. 2018;52:13–22. doi: 10.1016/j.bpobgyn.2018.01.022 29703554

[4] Pascal A, Govaert P, Oostra A, Naulaers G, Ortibus E, Van den Broeck C. Neurodevelopmental outcome in very preterm and very-low-birthweight infants born over the past decade: a meta-analytic review. *Dev Med Child Neurol*. 2018;60(4):342–355. doi: 10.1111/dmcn.13675 29350401

[5] Ely DM, Driscoll AK. Infant mortality in the United States, 2018: Data from the period linked birth/infant death file. National vital statistics reports vol 69 no 7. Hyattsville, MD: National Center for Health Statistics. 2020. 32730740

[6] Waitzman NJ, Jalali A, Grosse SD. Preterm birth lifetime costs in the United States in 2016: An update. *Semin Perinatol*, 2021; 45(3). doi: 10.1016/j.semperi.2021.151390 33541716 PMC10549985

[7] Burris HH, Riis VM, Schmidt I, Gerson KD, Brown A, Elovitz MA. Maternal stress, low cervicovaginal β-defensin, and spontaneous preterm birth. *Am J Obstet Gynecol MFM*. 2020 May;2(2):100092. doi: 10.1016/j.ajogmf.2020.100092 Epub 2020 Feb 10. . 32671334 PMC7363402

[8] Deguen S, Marchetta GP, Kihal-Talantikite W. Measuring Burden of Disease Attributable to Air Pollution Due to Preterm Birth Complications and Infant Death in Paris Using Disability-Adjusted Life Years (DALYs). Int J Environ Res Public Health. 2020;17(21):7841. doi: 10.3390/ijerph17217841 . 33114696 PMC7663522

[9] Root ED, Bailey ED, Gorham T, Browning C, Song C, Salsberry P. Geovisualization and Spatial Analysis of Infant Mortality and Preterm Birth in Ohio, 2008–2015: Opportunities to Enhance Spatial Thinking. *Public Health Rep*. 2020;135(4):472–482. doi: 10.1177/0033354920927854 32552459 PMC7383761

[10] Harron K, Verfuerden M, Ibiebele I, Liu C, Kopp A, Guttmann A, et al. Preterm birth, unplanned hospital contact, and mortality in infants born to teenage mothers in five countries: An administrative data cohort study. *Paediatr Perinat Epidemiol*. 2020;34(6):645–654. doi: 10.1111/ppe.12685 32343005 PMC8425326

[11] Opondo C, Gray R, Hollowell J, Li Y, Kurinczuk JJ, Quigley MA. Joint contribution of socioeconomic circumstances and ethnic group to variations in preterm birth, neonatal mortality and infant mortality in England and Wales: a population-based retrospective cohort study using routine data from 2006 to 2012. *BMJ Open*. 2019;9(7):e028227. doi: 10.1136/bmjopen-2018-028227 31371291 PMC6677942

[12] Soneji S, Beltrán-Sánchez H. Association of Special Supplemental Nutrition Program for Women, Infants, and Children With Preterm Birth and Infant Mortality. *JAMA Netw Open*. 2019;2(12):e1916722. doi: 10.1001/jamanetworkopen.2019.16722 31800070 PMC6902759

[13] Short VL, Hoffman M, Metgud M, Kavi A, Goudar SS, Okitawutshu J, et al. Safety of daily low-dose aspirin use during pregnancy in low-income and middle-income countries. *AJOG Glob Rep*. 2021 Feb;1(1):100003. doi: 10.1016/j.xagr.2021.100003 34085052 PMC8171270

[14] Burris HH, Wright CJ, Kirpalani H, Collins JW Jr, Lorch SA, Elovitz MA, et al. The promise and pitfalls of precision medicine to resolve black-white racial disparities in preterm birth. *Pediatr Res*. 2020;87(2):221–226. doi: 10.1038/s41390-019-0528-z 31382269

[15] Patki M, Giusto K, Gorasiya S, Reznik SE, Patel K. 17-α Hydroxyprogesterone Nanoemulsifying Preconcentrate-Loaded Vaginal Tablet: A Novel Non-Invasive Approach for the Prevention of Preterm Birth. *Pharmaceutics*. 2019;11(7):335. 10.3390/pharmaceutics11070335 PMC6680947 31337153

[16] MacDorman MF, Declercq E, Zhang J. Obstetrical intervention and the singleton preterm birth rate in the United States from 1991–2006. *Am J Public Health*. 2010;100(11):2241–7. 20864720 10.2105/AJPH.2009.180570 PMC2951941

[17] National Center for Health Statistics. User Guide to the 2018 Natality Public-Use File. Hyattsville, MD: National Center for Health Statistics. ftp://ftp.cdc.gov/pub/Health_Statistics/NCHS/Dataset_Documentation/DVS/natality/UserGuide2018-508.pdf. Accessed July 1, 2021.

[18] Martin JA, Osterman MJK, Kirmeyer SE, Gregory ECW. Measuring gestational age in vital statistics data. Transitioning to the obstetric estimate. *National vital statistics reports* vol 64 no 5. Hyattsville, MD: National Center for Health Statistics. 2015. 26047089

[19] National Center for Health Statistics. U.S. Standard Certificate of Live Birth. https://www.cdc.gov/nchs/data/dvs/birth11-03final-acc.pdf. Accessed July 1, 2021.

[20] American College of Obstetricians and Gynecologists. ACOG Committee Opinion #831. Medically indicated late-preterm and early-term deliveries. *Obstet Gynecol* 2021; 138(1): e35–e39. 34259491 10.1097/AOG.0000000000004447

[21] California Maternal Quality Care Collaborative (CMQCC). *Elimination of non-medically indicated (Elective) deliveries before 39 weeks gestational age*: *A California toolkit to transform maternity care*. 2010: CMQCC. https://www.cmqcc.org/resources-tool-kits/toolkits/early-elective-deliveries-toolkit. Accessed: August 1, 2021.

[22] Jordana Frost, Director, Strategic Partnerships, March of Dimes, personal communication (email). July 19, 2021.

[23] Bettegowda VR, Dias T, Davidoff MJ, Damus K, Callaghan WM, Petrini JR. The relationship between cesarean delivery and gestational age among US singleton births. *Clin Perinatol*. 2008;35(2):309–23, v–vi. doi: 10.1016/j.clp.2008.03.002 18456071

[24] The joint commission. Specifications Manual for Joint Commission National Quality Measures. American Medical Association. 2021. https://manual.jointcommission.org/releases/archive/pdf_archive/TJC_v2021A.pdf. Accessed September 24, 2021.

[25] Auger N, Luo ZC, Nuyt AM, Kaufman JS, Naimi AI, Platt RW, et al. Secular trends in preeclampsia incidence and outcomes in a large Canada database. A longitudinal study over 24 years. *Canadian J Cardiology*. 2016; 32(8): 987. 10.1016/j.cjca.2015.12.011 26947535

[26] Thompson J, Onyenake C, Oduguwa DD, Gendra S, Coker V, et al. Trends and Racial/Ethnic Disparities in the Rates of Pre-eclampsia by HIV Status in the US. *J Racial Ethn Health Disparities*. 2021;8(3):670–677. doi: 10.1007/s40615-020-00826-3 32754847

[27] Bryant AS, Worjoloh A, Caughey AB, Washington AE. Racial/ethnic disparities in obstetric outcomes and care: prevalence and determinants. *Am J Obstet Gynecol*. 2010;202(4):335–43. doi: 10.1016/j.ajog.2009.10.864 20060513 PMC2847630

[28] Howell EA, Egorova NN, Balbierz A, Zeitlin J, Hebert PL. Site of delivery contribution to black-white severe maternal morbidity disparity. *Am J Obstet Gynecol*. 2016;215(2):143–52. doi: 10.1016/j.ajog.2016.05.007 27179441 PMC4967380

[29] Howell EA, Egorova N, Balbierz A, Zeitlin J, Hebert PL. Black-white differences in severe maternal morbidity and site of care. *Am J Obstet Gynecol*. 2016;214(1):122.e1–7. doi: 10.1016/j.ajog.2015.08.019 26283457 PMC4698019

[30] Grobman WA, Baillit JL, Rice MM, Wapner RJ, Reddy U, et al. Racial and ethnic disparities in maternal morbidity and obstetric care. *Obstet Gynecol* 2015: 1245(6): 1450–7. doi: 10.1097/AOG.0000000000000735 26000518 PMC4443856

[31] Janevic T, Piverger N, Afzal O, Howell EA. "Just Because You Have Ears Doesn’t Mean You Can Hear"-Perception of Racial-Ethnic Discrimination During Childbirth. *Ethn Dis*. 2020;30(4):533–542. doi: 10.18865/ed.30.4.533 32989353 PMC7518531

[32] Hamm RF, Srinivas SK, Levine LD. A standardized labor induction protocol: impact on racial disparities in obstetrical outcomes. *Am J Obstet Gynecol MFM*. 2020;2(3):100148. doi: 10.1016/j.ajogmf.2020.100148 33345879

[33] Gregory ECW, Martin JA, Argov EL, Osterman MJK. Assessing the quality of medical and health data from the 2003 birth certificate revision: Results from New York City. *Natl Vital Stat Rep*. 2019; 68(8): 1–20. 32501201

[34] Martin JA, Wilson EC, Osterman MJK, Saadi EW, Sutton SR, Hamilton BE. Assessing the quality of medical and health data from the 2003 birth certificate revision: Results from two states. *Vital Health Stat* 2013; 62(2): 1–20. 24979975

[35] Wise LA, Wang TR, Wesselink AK, Willis SK, et al. Accuracy of self-reported birth outcomes relative to birth certificate data in an Internet-based prospective cohort study. *Paediatr Perinat Epidemiol* May 6, 2021 published online ahead of print. 10.1111/ppe.12769 PMC8380669 33956369

[36] Kane DJ, Sappenfield WM. Ascertainment of Medicaid payment for delivery on the Iowa birth certificate: Is accuracy sufficient for timely policy and program relevant analysis? *Matern Child Health J*. 2014; 18(4): 970–7. doi: 10.1007/s10995-013-1325-7 23832375

[37] Stout MJ, Macones GA, Tuuli MG. Accuracy of birth certificate data for classifying preterm birth. Paediatr Perinat Epidemiol. 2017; 31(3): 245–9. doi: 10.1111/ppe.12352 28370345

[38] Haghighat N, Hu M, Laurent O, Chung J, Nguyen P Wu J. Comparison of birth certificates and hospital-based birth data on pregnancy complications in Los Angeles and Orange County, California. BMC pregnancy childbirth 2016; 16:93. doi: 10.1186/s12884-016-0885-0 27121857 PMC4848813

[39] Dietz P, Bombard J. Mulready-Ward C, Gauthier J, et al. Validation of selected items on the 2003 U.S. standard certificate of live birth: New York City and Vermont. Public Health Rep. 2015; 130(1): 60–70. doi: 10.1177/003335491513000108 25552756 PMC4245286

[40] Martin JA, Hamilton BE, Sutton P 418 D, Ventura SJ, et al. Births: Final data for 2006. National vital statistics reports; vol 57 no 7. Hyattsville, MD: National Center for Health Statistics. 2009.

[41] White VanGompel E, Perez S, Datta A, Wang C, Cape V, Main E. Cesarean overuse and the culture of care. *Health Serv Res*. 2019;54(2):417–424. doi: 10.1111/1475-6773.13123 30790273 PMC6407356

